# Supplementary material for: Individual adherence to mass drug administration in neglected tropical disease control: A probability model conditional on past behaviour
Source: PLoS Negl Trop Dis. 2021 Jan 22;15(1):e0009112. doi: 10.1371/journal.pntd.0009112 (PMC7857569; doi:10.1371/journal.pntd.0009112)
Supplement: S1 Appendix — (PDF) [file pntd.0009112.s001.pdf]

S1 Appendix.

Summary. In this supplementary information we derive the key mathematical expressions which are used and referred to in the main text.

Time-independent Markov model

Assuming that the conditional probabilities β and γ are constant, the time-independent Markov model may be mapped to the following recursion relation

$$p_n = \beta p_{n-1} + (1-\gamma)(1-p_{n-1}) \,. \tag{1}$$

As in the main text, defining the system state vector as

$$\mathbf{p}_n = \begin{pmatrix} p_n \\ 1-p_n \end{pmatrix} \,, \tag{2}$$

we may rewrite Eq (1) above in the form $\mathbf{p}_n = \mathbf{M}\,\mathbf{p}_{n-1}$ where we have defined the following transition matrix

$$\mathbf{M} \equiv \begin{pmatrix} \beta & 1-\gamma \\ 1-\beta & \gamma \end{pmatrix} \,. \tag{3}$$

The eigenvalues and eigenvectors of $\mathbf{M}$ are given by

$$\lambda' = 1 \,, \quad \mathbf{v}' = \frac{1}{2-\gamma-\beta}\begin{pmatrix} 1-\gamma \\ 1-\beta \end{pmatrix} \equiv \begin{pmatrix} q \\ 1-q \end{pmatrix} \,, \tag{4}$$

$$\lambda = \beta+\gamma-1 \,, \quad \mathbf{v} = \begin{pmatrix} -1 \\ 1 \end{pmatrix} \,. \tag{5}$$

where $\mathbf{v}'$ is normalised to sum to 1. Given that $|\lambda| < 1$ in all realistic circumstances, it is clear from this description that $\mathbf{v}$ represents the equilibrium of the system over multiple rounds with λ defining the rate of relaxation towards it. When $\lambda = 0$, the model becomes a history-independent model in which the next round is dictated solely by its probability at that round.

In order to study the dynamics in more detail, we apply the following transformation

$$p_n \to \tilde{p}_n = p_n(\beta+\gamma-1)^{1-n} \,, \tag{6}$$

to the relation given by Eq (1), such that

$$\tilde{p}_n = \tilde{p}_{n-1} + (1-\gamma)(\beta+\gamma-1)^{1-n} \,. \tag{7}$$

Through explicit summation, Eq (7) is solved by

$$\tilde{p}_n - \tilde{p}_1 = \sum_{n'=2}^{n} (\tilde{p}_{n'} - \tilde{p}_{n'-1}) = \sum_{n'=2}^{n} (1-\gamma)(\beta+\gamma-1)^{1-n'} \,. \tag{8}$$

By reapplying the inverse transformation $\tilde{p}_n \to p_n$ to Eq (8) and identifying $\tilde{p}_1 = p_1 = \alpha$, we obtain the following solution to Eq (1)

$$\begin{aligned} p_n &= \alpha(\beta+\gamma-1)^{n-1} + \sum_{n'=2}^{n} (1-\gamma)(\beta+\gamma-1)^{n-n'} \\ &= \alpha(\beta+\gamma-1)^{n-1} + \frac{1-\gamma}{\beta+\gamma-2}\left[(\beta+\gamma-1)^{n-1} - 1\right] \,. \end{aligned} \tag{9}$$

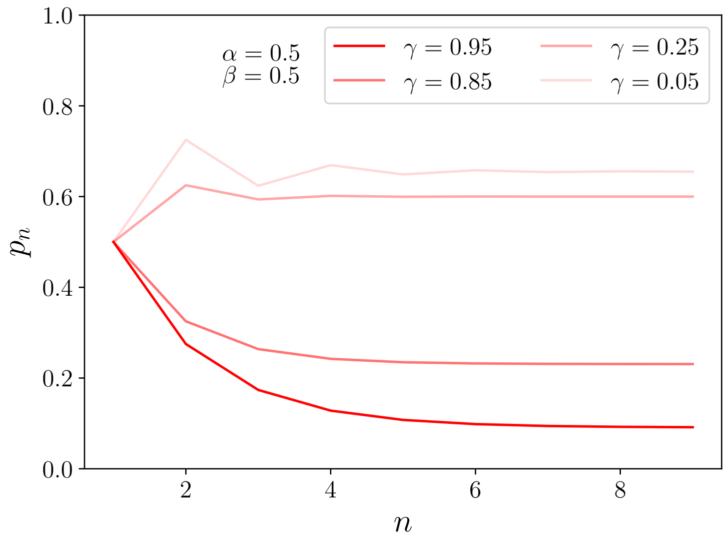

Fig a. The probability of receiving treatment in the n-th round given by the Markovian model solution in Eq (9) for a range of γ values. The other probabilities have been fixed to $\alpha = 0.5$ and $\beta = 0.5$.

Equivalently, satisfying the dual to Eq (1) in terms of the probability of non-treatment in the n-th round $1 - p_n$, solutions to Eq (9) must also satisfy

$$1 - p_n = (1-\alpha)(\beta + \gamma - 1)^{n-1} + \frac{1-\beta}{\beta+\gamma-2}\left[(\beta+\gamma-1)^{n-1} - 1\right] . \qquad (10)$$

In Fig a we illustrate the dynamics of the system using Eq (9) with range of parameter values chosen for γ. Notice, in particular, that the system exhibits oscillation before relaxing to a steady state when γ is chosen such that the eigenvalue $\lambda = \beta + \gamma - 1 < 0$.

For another way of calculating the expected lengths of repeat adherence $\mathrm{E}(n_{\mathrm{T}})$ or non-adherence $\mathrm{E}(n_{\mathrm{F}})$ of an individual (as computed in the main text), given that they begin with the same choice in the first round, one need only fix $(\alpha = \beta,\, \gamma = 1)$ or $(\alpha = 1 - \gamma,\, \beta = 1)$ and take moments with Eq (9), respectively, such that

$$\begin{aligned}(\alpha = \beta,\, \gamma = 1) \;\Rightarrow\; \mathrm{E}(n_{\mathrm{T}}) &= \sum_{n=0}^{\infty} n\left(1 - \frac{p_n}{p_{n-1}}\right)p_{n-1} \\ &= \sum_{n=0}^{\infty} n(1-\beta)\beta^{n-1} = \frac{1}{1-\beta} \qquad (11)\\ (\alpha = 1-\gamma,\, \beta = 1) \;\Rightarrow\; \mathrm{E}(n_{\mathrm{F}}) &= \sum_{n=0}^{\infty} n\left(1 - \frac{1-p_n}{1-p_{n-1}}\right)(1-p_{n-1}) \\ &= \sum_{n=0}^{\infty} n(1-\gamma)\gamma^{n-1} = \frac{1}{1-\gamma} . \qquad (12)\end{aligned}$$

Time-dependent Markov model

Consider the choice matrices with elements $C^{\mathrm{T}}_{nn'}$ and $C^{\mathrm{F}}_{nn'}$ corresponding to the conditional probabilities of treatment and non-treatment in round n given treatment

and non-treatment in round n', respectively, such that

$$p_n = \sum_{n'=1}^{n-1} \left[C^{\mathrm{T}}_{nn'} p_{n'} + C^{\mathrm{F}}_{nn'} (1 - p_{n'}) \right] \,. \tag{13}$$

When the only nonzero elements of the choice matrices in Eq (13) are along the their lower diagonals, i.e., such that only $C^{\mathrm{T}}_{n\,n-1} = \beta_{n\,n-1} \neq 0$ and $C^{\mathrm{F}}_{n\,n-1} = 1 - \gamma_{n\,n-1} \neq 0$, the system is described by a time-dependent Markov process with recursion relation

$$p_n = \beta_{n\,n-1} p_{n-1} + (1 - \gamma_{n\,n-1})(1 - p_{n-1}) \,. \tag{14}$$

Following a similar argument to the one used in solving the homogeneous Markov case, we may obtain an implicit solution to Eq (14). Using the transformation

$$p_n \to \tilde{p}_n = \frac{p_n}{\prod_{n'=2}^{n} (\beta_{n'n'-1} + \gamma_{n'n'-1} - 1)} \,, \tag{15}$$

we once again substitute into the relation given by Eq (14), yielding

$$\tilde{p}_n = \tilde{p}_{n-1} + \frac{1 - \gamma_{n\,n-1}}{\prod_{n'=2}^{n} (\beta_{n'n'-1} + \gamma_{n'n'-1} - 1)} \,, \tag{16}$$

where Eq (16) is solved by the explicit summation

$$\tilde{p}_n - \tilde{p}_1 = \sum_{n''=2}^{n} (\tilde{p}_{n''} - \tilde{p}_{n''-1}) = \sum_{n''=2}^{n} \frac{1 - \gamma_{n''n''-1}}{\prod_{n'=2}^{n''} (\beta_{n'n'-1} + \gamma_{n'n'-1} - 1)} \,. \tag{17}$$

Using the corresponding inverse transformation to Eq (16) we hence obtain a solution to Eq (14), which is given by

$$p_n = \alpha \prod_{n'=2}^{n} (\beta_{n'n'-1} + \gamma_{n'n'-1} - 1) + \sum_{n''=2}^{n} (1 - \gamma_{n''n''-1}) \prod_{n'=n''}^{n} (\beta_{n'n'-1} + \gamma_{n'n'-1} - 1) \,. \tag{18}$$

General choice matrices: non-Markovian models

The most general set of causal adherence models described by Eq (13) have choice matrices which take the form

$$\boldsymbol{C}^{\mathrm{T}} = \begin{pmatrix} 0 & 0 & 0 & \dots \\ C^{\mathrm{T}}_{n\,n-1} & 0 & 0 & \dots \\ C^{\mathrm{T}}_{n\,n-2} & C^{\mathrm{T}}_{n-1\,n-2} & 0 & \dots \\ \vdots & \vdots & \vdots & \end{pmatrix} \qquad \boldsymbol{C}^{\mathrm{F}} = \begin{pmatrix} 0 & 0 & 0 & \dots \\ C^{\mathrm{F}}_{n\,n-1} & 0 & 0 & \dots \\ C^{\mathrm{F}}_{n\,n-2} & C^{\mathrm{F}}_{n-1\,n-2} & 0 & \dots \\ \vdots & \vdots & \vdots & \end{pmatrix} \,, \tag{19}$$

where 'non-Markovian' behaviour in the n-th round clearly corresponds to a past behaviour dependence between rounds which exceeds the immediate last round, i.e., $C^{\mathrm{T,F}}_{n\,n-m} \neq 0$ where $m > 1$.

Notice that all of the adherence models that we have identified in this work may be categorised by various constraints on the elements of the choice matrices introduced in Eq (13). For completeness and reference, these are

1. Past behaviour-independent adherence that is time-independent: $\forall n > 1$ only $C^{\mathrm{T,F}}_{n\,n-1} \neq 0$, $C^{\mathrm{T}}_{n\,n-1} = C^{\mathrm{F}}_{n\,n-1} = c$ and $p_1 = c$, giving one degree of freedom multiplied by the number of independent bins for population-level heterogeneity.

2. Past behaviour-independent adherence that is time-dependent: $\forall n > 1$ only $C^{\mathrm{T,F}}_{n\,n-1} \neq 0$, $C^{\mathrm{T}}_{n\,n-1} = C^{\mathrm{F}}_{n\,n-1} = c_n$ and $p_1 = c_1$, giving n degrees of freedom multiplied by the number of independent bins for population-level heterogeneity.

3. Markovian past behaviour-dependent adherence that is time-independent: $\forall n > 1$ only $C^{\mathrm{T,F}}_{n\,n-1} \neq 0$, $C^{\mathrm{T}}_{n\,n-1} = \beta$, $C^{\mathrm{F}}_{n\,n-1} = 1-\gamma$ and $p_1 = \alpha$, giving 3 degrees of freedom multiplied by the number of independent bins for population-level heterogeneity.

4. Markovian past behaviour-dependent adherence that is time-dependent: $\forall n > 1$ only $C^{\mathrm{T,F}}_{n\,n-1} \neq 0$, $C^{\mathrm{T}}_{n\,n-1} = \beta_{n\,n-1}$, $C^{\mathrm{F}}_{n\,n-1} = 1-\gamma_{n\,n-1}$ and $p_1 = \alpha$, giving $2n-1$ degrees of freedom multiplied by the number of independent bins for population-level heterogeneity.

5. Non-Markovian past behaviour-dependent adherence that is time-dependent: $\forall n > 1$ and $\forall n' < n$ only $C^{\mathrm{T,F}}_{nn'} \neq 0$ and $p_1 = \alpha$, giving $1 + n(n-1)$ degrees of freedom multiplied by the number of independent bins for population-level heterogeneity.

Likelihoods and Bayesian evidence

Let the data now correspond to a set of n-vectors $D = \{\boldsymbol{X}\}$ where each individual's adherence or non-adherence behaviour in the n-th round is recorded, such that $X_n = \mathrm{T}, \mathrm{F}$. Using Eq (13) the full generalisation of the likelihood (which supports all of the possible adherence models, becomes

$$\mathcal{L}(D|\boldsymbol{\theta}) = \prod_{\forall X_n \in D} \prod_{n'=1}^{n} \left\{ \sum_{n''=1}^{n'-1} \left[C^{\mathrm{T}}_{nn'} \mathbb{1}_{X_{n'}=\mathrm{T}} + C^{\mathrm{F}}_{nn'} \mathbb{1}_{X_{n'}=\mathrm{F}} \right] \right\}, \tag{20}$$

where $\mathbb{1}_A$ denotes an indicator function which takes value unity when condition A is satisfied, else it vanishes.

The large number of available degrees of freedom in Eq (20) motivates a systematic approach to inferring the choice matrix components from a given set of data. We elect to consider models which isolate the many degrees of freedom by constructing scenarios where past behaviour-dependent adherence only occurs for a single round and is temporally dependent on only one other round — all other degrees of freedom are hence set to those corresponding to time-dependent past behaviour-independent adherence, i.e. $C^{\mathrm{T}}_{nn'} = C^{\mathrm{F}}_{nn'} = c_n$. The likelihood for this more restricted set of models — which we denote as $\mathcal{L}_{nn'}(D|\boldsymbol{\theta})$, where nn' corresponds to the pair of rounds chosen to be dependent on each other in time — may be obtained by rewriting Eq (20) in the following form

$$\mathcal{L}_{nn'}(D|\boldsymbol{\theta}) = \\ (1-C^{\mathrm{T}}_{nn'})^{Z^{n'n}_{\mathrm{TF}}} (C^{\mathrm{T}}_{nn'})^{Z^{n'n}_{\mathrm{TT}}} (1-C^{\mathrm{F}}_{nn'})^{Z^{n'n}_{\mathrm{FF}}} (C^{\mathrm{F}}_{nn'})^{Z^{n'n}_{\mathrm{FT}}} \prod_{\forall n'' \neq n} c_{n''}^{N_{n''}} (1-c_{n''})^{N-N_{n''}}\,, \tag{21}$$

where we have defined

$$Z^{n'n}_{\mathrm{AB}} \equiv \sum_{\{\forall \boldsymbol{X} \,|\, X_{n'}=\mathrm{A},\, X_n=\mathrm{B}\}} N_{\boldsymbol{X}}\,, \tag{22}$$

where the data $D = \{N_{\boldsymbol{X}}\}$ has now been compressed into the set of numbers of people who track the same behaviour as $\boldsymbol{X}$, i.e., for 3 rounds, this forms the set of the

following numbers of people: $N_{\mathrm{TTT}}, N_{\mathrm{TTF}}, N_{\mathrm{TFT}}$, etc. The Bayesian evidence integral corresponding to Eq (21) with a choice of flat prior $\pi(\boldsymbol{\theta}) \propto 1$ is therefore

$$\begin{aligned}\mathcal{E}_{nn'} &= \int_0^1 (1 - C^{\mathrm{T}}_{nn'})^{Z^{n'n}_{\mathrm{TF}}} (C^{\mathrm{T}}_{nn'})^{Z^{n'n}_{\mathrm{TT}}} \int_0^1 (1 - C^{\mathrm{F}}_{nn'})^{Z^{n'n}_{\mathrm{FF}}} (C^{\mathrm{F}}_{nn'})^{Z^{n'n}_{\mathrm{FT}}} \mathrm{d}C^{\mathrm{T}}_{nn'} \mathrm{d}C^{\mathrm{F}}_{nn'} \\ &\quad \times \prod_{\forall n'' \neq n} \left[\int_0^1 c_{n''}^{N_{n''}} (1 - c_{n''})^{N - N_{n''}} \mathrm{d}c_{n''} \right] \\ &= \frac{\Gamma(Z^{n'n}_{\mathrm{TF}} + 1)\Gamma(Z^{n'n}_{\mathrm{TT}} + 1)}{\Gamma(Z^{n'n}_{\mathrm{TT}} + Z^{n'n}_{\mathrm{TF}} + 2)} \frac{\Gamma(Z^{n'n}_{\mathrm{FF}} + 1)\Gamma(Z^{n'n}_{\mathrm{FT}} + 1)}{\Gamma(Z^{n'n}_{\mathrm{FF}} + Z^{n'n}_{\mathrm{FT}} + 2)} \\ &\quad \times \prod_{\forall n'' \neq n} \frac{\Gamma(N_{n''} + 1)\Gamma(N - N_{n''} + 1)}{\Gamma(N + 2)} . \end{aligned} \tag{23}$$

Some non-Markovian past dependence may be captured by the likelihood defined in Eq (21), however their Bayesian evidence may need to be compared with equivalent Markovian models which also generate decaying long-term correlations of a particular form. Using the same formalism as Eq (21), the time-dependent Markov model has the following likelihood

$$\begin{aligned}&\mathcal{L}(D|\boldsymbol{\theta}) = \\ &\alpha^{N_{\mathrm{T}}}(1-\alpha)^{N_{\mathrm{F}}} \prod_{\forall n \geq 2} (1 - C^{\mathrm{T}}_{n\,n-1})^{Z^{n-1\,n}_{\mathrm{TF}}} (C^{\mathrm{T}}_{n\,n-1})^{Z^{n-1\,n}_{\mathrm{TT}}} (1 - C^{\mathrm{F}}_{n\,n-1})^{Z^{n-1\,n}_{\mathrm{FF}}} (C^{\mathrm{F}}_{n\,n-1})^{Z^{n-1\,n}_{\mathrm{FT}}} ,\end{aligned} \tag{24}$$

and, hence, yields the following Bayesian evidence

$$\begin{aligned}\mathcal{E} &= \int_0^1 \alpha^{N_{\mathrm{T}}}(1-\alpha)^{N_{\mathrm{F}}} \mathrm{d}\alpha \prod_{\forall n \geq 2} \int_0^1 (1 - C^{\mathrm{T}}_{n\,n-1})^{Z^{n-1\,n}_{\mathrm{TF}}} (C^{\mathrm{T}}_{n\,n-1})^{Z^{n-1\,n}_{\mathrm{TT}}} \\ &\quad \times \int_0^1 (1 - C^{\mathrm{F}}_{n\,n-1})^{Z^{n-1\,n}_{\mathrm{FF}}} (C^{\mathrm{F}}_{n\,n-1})^{Z^{n-1\,n}_{\mathrm{FT}}} \mathrm{d}C^{\mathrm{T}}_{n\,n-1} \mathrm{d}C^{\mathrm{F}}_{n\,n-1} \\ &= \frac{\Gamma(N_{\mathrm{T}}+1)\Gamma(N_{\mathrm{F}}+1)}{\Gamma(N+2)} \prod_{\forall n \geq 2} \frac{\Gamma(Z^{n-1\,n}_{\mathrm{TF}} + 1)\Gamma(Z^{n-1\,n}_{\mathrm{TT}} + 1)}{\Gamma(Z^{n-1\,n}_{\mathrm{TT}} + Z^{n-1\,n}_{\mathrm{TF}} + 2)} \frac{\Gamma(Z^{n-1\,n}_{\mathrm{FF}} + 1)\Gamma(Z^{n-1\,n}_{\mathrm{FT}} + 1)}{\Gamma(Z^{n-1\,n}_{\mathrm{FF}} + Z^{n-1\,n}_{\mathrm{FT}} + 2)} .\end{aligned} \tag{25}$$

Eqs (24) and (25) may also be used to obtain the likelihood of the time-independent Markov model

$$\begin{aligned}&\mathcal{L}(D|\boldsymbol{\theta}) = \\ &\alpha^{N_{\mathrm{T}}}(1-\alpha)^{N_{\mathrm{F}}} \beta^{\sum_{\forall n \geq 2} Z^{n-1\,n}_{\mathrm{TT}}} (1-\beta)^{\sum_{\forall n \geq 2} Z^{n-1\,n}_{\mathrm{TF}}} \gamma^{\sum_{\forall n \geq 2} Z^{n-1\,n}_{\mathrm{FF}}} (1-\gamma)^{\sum_{\forall n \geq 2} Z^{n-1\,n}_{\mathrm{FT}}} ,\end{aligned} \tag{26}$$

and the Bayesian evidence of the same model

$$\mathcal{E} = \int_0^1 \alpha^{N_\mathrm{T}}(1-\alpha)^{N_\mathrm{F}}\mathrm{d}\alpha \int_0^1 \beta^{\sum_{\forall n\geq 2} Z_{\mathrm{TT}}^{n-1\,n}}(1-\beta)^{\sum_{\forall n\geq 2} Z_{\mathrm{TF}}^{n-1\,n}}\mathrm{d}\beta$$
$$\times \int_0^1 \gamma^{\sum_{\forall n\geq 2} Z_{\mathrm{FF}}^{n-1\,n}}(1-\gamma)^{\sum_{\forall n\geq 2} Z_{\mathrm{FT}}^{n-1\,n}}\mathrm{d}\gamma$$
$$= \frac{\Gamma(N_\mathrm{T}+1)\Gamma(N_\mathrm{F}+1)}{\Gamma(N+2)} \frac{\Gamma\left(\sum_{\forall n\geq 2} Z_{\mathrm{TF}}^{n-1\,n}+1\right)\Gamma\left(\sum_{\forall n\geq 2} Z_{\mathrm{TT}}^{n-1\,n}+1\right)}{\Gamma\left[\sum_{\forall n\geq 2}\left(Z_{\mathrm{TT}}^{n-1\,n}+Z_{\mathrm{TF}}^{n-1\,n}\right)+2\right]}$$
$$\times \frac{\Gamma\left(\sum_{\forall n\geq 2} Z_{\mathrm{FF}}^{n-1\,n}+1\right)\Gamma\left(\sum_{\forall n\geq 2} Z_{\mathrm{FT}}^{n-1\,n}+1\right)}{\Gamma\left[\sum_{\forall n\geq 2}\left(Z_{\mathrm{FF}}^{n-1\,n}+Z_{\mathrm{FT}}^{n-1\,n}\right)+2\right]}. \quad (27)$$

References

1. Boatin BA, Basáñez MG, Prichard RK, Awadzi K, Barakat RM, García HH, et al. A research agenda for helminth diseases of humans: towards control and elimination. PLoS neglected tropical diseases. 2012;6(4):e1547. Available from: https://doi.org/10.1371/journal.pntd.0001547.

2. Krentel A, Fischer PU, Weil GJ. A review of factors that influence individual compliance with mass drug administration for elimination of lymphatic filariasis. PLoS neglected tropical diseases. 2013;7(11):e2447. Available from: https://doi.org/10.1371/journal.pntd.0002447.

3. Truscott JE, Hollingsworth TD, Brooker SJ, Anderson RM. Can chemotherapy alone eliminate the transmission of soil transmitted helminths? Parasites & vectors. 2014;7(1):266. Available from: https://doi.org/10.1186/1756-3305-7-266.

4. Babu BV, Babu GR. Coverage of, and compliance with, mass drug administration under the programme to eliminate lymphatic filariasis in India: a systematic review. Transactions of the Royal Society of Tropical Medicine and Hygiene. 2014;108(9):538–549. Available from: https://doi.org/10.1093/trstmh/tru057.

5. Irvine MA, Reimer LJ, Njenga SM, Gunawardena S, Kelly-Hope L, Bockarie M, et al. Modelling strategies to break transmission of lymphatic filariasis - aggregation, adherence and vector competence greatly alter elimination. Parasites & Vectors. 2015 Oct;8(1):547. Available from: https://doi.org/10.1186/s13071-015-1152-3.

6. Coffeng LE, Bakker R, Montresor A, de Vlas SJ. Feasibility of controlling hookworm infection through preventive chemotherapy: a simulation study using the individual-based WORMSIM modelling framework. Parasites & vectors. 2015;8(1):541. Available from: https://doi.org/10.1186/s13071-015-1151-4.

7. Shuford KV, Turner HC, Anderson RM. Compliance with anthelmintic treatment in the neglected tropical diseases control programmes: a systematic review. Parasites & vectors. 2016;9(1):29. Available from: https://doi.org/10.1186/s13071-016-1311-1.

8. Farrell SH, Truscott JE, Anderson RM. The importance of patient compliance in repeated rounds of mass drug administration (MDA) for the elimination of intestinal helminth transmission. Parasites & Vectors. 2017 Jun;10(1):291. Available from: https://doi.org/10.1186/s13071-017-2206-5.

9. World Health Organization. Guideline: preventive chemotherapy to control soil-transmitted helminth infections in at-risk population groups. World Health Organization; 2017.

10. Fogarty L, Roter D, Larson S, Burke J, Gillespie J, Levy R. Patient adherence to HIV medication regimens: a review of published and abstract reports. Patient Education and Counseling. 2002;46(2):93 – 108. Available from: http://www.sciencedirect.com/science/article/pii/S0738399101002191.

11. Pullan RL, Halliday KE, Oswald WE, Mcharo C, Beaumont E, Kepha S, et al. Effects, equity, and cost of school-based and community-wide treatment strategies for soil-transmitted helminths in Kenya: a cluster-randomised controlled trial. The Lancet. 2019;393(10185):2039–2050. Available from: https://doi.org/10.1016/S0140-6736(18)32591-1.

12. Halliday KE, Oswald WE, Mcharo C, Beaumont E, Gichuki PM, Kepha S, et al. Community-level epidemiology of soil-transmitted helminths in the context of school-based deworming: Baseline results of a cluster randomised trial on the coast of Kenya. PLoS neglected tropical diseases. 2019;13(8):e0007427. Available from: https://doi.org/10.1371/journal.pntd.0007427.

13. Oswald WE, Kepha S, Halliday KE, Mcharo C, Witek-McManus S, Hardwick RJ, et al. Patterns of individual non-treatment during multiple rounds of mass drug administration for control of soil-transmitted helminths in the TUMIKIA trial, Kenya: a secondary longitudinal analysis. The Lancet Global Health. 2020;8(11):e1418–e1426. Available from: https://doi.org/10.1016/S2214-109X(20)30344-2.

14. Plaisier AP. Modelling onchocerciasis transmission and control. Erasmus University Rotterdam; 1996.

15. Plaisier AP, Stolk WA, van Oortmarssen GJ, Habbema JDF. Effectiveness of annual ivermectin treatment for Wuchereria bancrofti infection. Parasitology Today. 2000;16(7):298–302. Available from: https://doi.org/10.1016/s0169-4758(00)01691-4.

16. Jeffreys H. The theory of probability. OUP Oxford; 1998.

17. Truscott JE, Ower AK, Werkman M, Halliday K, Oswald WE, Gichuki PM, et al. Heterogeneity in transmission parameters of hookworm infection within the baseline data from the TUMIKIA study in Kenya. Parasites & vectors. 2019;12(1):442. Available from: https://doi.org/10.1186/s13071-019-3686-2.

18. Ásbjörnsdóttir KH, Ajjampur SSR, Anderson RM, Bailey R, Gardiner I, Halliday KE, et al. Assessing the feasibility of interrupting the transmission of soil-transmitted helminths through mass drug administration: The DeWorm3 cluster randomized trial protocol. PLOS Neglected Tropical Diseases. 2018 01;12(1):1–16. Available from: https://doi.org/10.1371/journal.pntd.0006166.

19. Mekete K, Ower A, Dunn J, Sime H, Tadesse G, Abate E, et al. The Geshiyaro Project: a study protocol for developing a scalable model of interventions for moving towards the interruption of the transmission of soil-transmitted helminths and schistosome infections in the Wolaita zone of Ethiopia. Parasites & vectors. 2019;12(1):1–12. Available from: https://doi.org/10.1186/s13071-019-3757-4.

20. Dyson L, Stolk WA, Farrell SH, Hollingsworth TD. Measuring and modelling the effects of systematic non-adherence to mass drug administration. Epidemics. 2017;18:56–66. Available from: http://www.ncbi.nlm.nih.gov/pubmed/28279457http://www.pubmedcentral.nih.gov/articlerender.fcgi?artid=PMC5340860.

21. Griffin JT, Hollingsworth TD, Okell LC, Churcher TS, White M, Hinsley W, et al. Reducing Plasmodium falciparum Malaria Transmission in Africa: A Model-Based Evaluation of Intervention Strategies. PLoS Medicine. 2010 aug;7(8):e1000324. Available from: http://dx.plos.org/10.1371/journal.pmed.1000324.
