# Supplementary material for: Individual adherence to mass drug administration in neglected tropical disease control: A probability model conditional on past behaviour
Source: PLoS Negl Trop Dis. 2021 Jan 22;15(1):e0009112. doi: 10.1371/journal.pntd.0009112 (PMC7857569; doi:10.1371/journal.pntd.0009112)
Supplement: S2 Appendix — (PDF) [file pntd.0009112.s002.pdf]

S2 Appendix.

Summary. In this supplementary information we apply the framework of our mathematical model for adherence to the TUMIKIA project [11–13] and write a brief analysis description for each age group and sex.

Introduction

In Figs a, b, c, d and e we plot the maximum likelihood as well as the limits of the marginalised 95% credible region for the conditional probabilities given treatment (filled points) or non-treatment (hollow points) in a previous round of the overall, male and female participants in the top, middle and bottom rows, respectively. In the left column the constant conditional probabilities between any given sequential pair of rounds have been inferred, which corresponds to the time-independent Markov model of the main text and Appendix S1. In the right column all possible round pair dependencies are considered (indicated by the arrows on the horizontal axis), where in each case the components corresponding to a given round were measured assuming all other respective rounds were inferred to be from past behaviour-independent adherence. In all plots, above each pair of components we have also provided the log-Bayes factors [16], defined by

$$\ln \mathrm{B}_{nn'} = \ln \left(\frac{\mathcal{E}_{nn'}}{\mathcal{E}_{\mathrm{ref}}} \right) , \tag{1}$$

where the evidence for each pair $\mathcal{E}_{nn'}$ has been evaluated using the relations provided in Appendix S1 and the reference model evidence $\mathcal{E}_{\mathrm{ref}}$ has been set to that of time-dependent past behaviour-independent adherence for all components.

Results

In Figs a, b and c we present our results for the pre-SAC, SAC and 15-29 age groups of individuals in the TUMIKIA project. These age groups appear to be well-described by a time-dependent Markov model so past behaviour-dependent non-adherence is clearly present. This may be identified by the largest log-Bayes factor values being given in the red-coloured right column plots for all three sets of plots. However, the conditional probabilities in all groups appear to drift closer together by round 4 of treatment, which signals a gradual transition from past behaviour-dependent to independent adherence.

In Figs d and e we present our results for the 30-49 and 50+ age groups of individuals in the TUMIKIA project. The overall cohort, as well as the males and females in both age groups, appear to exhibit strong evidence of past behaviour-dependent non-adherence — in particular, they are all apparently well-described by a time-independent Markov model. These conclusions may be drawn both by the consistent distance between all of the values for the inferred conditional probabilities with the red points of the right column of plots, as well as the largest evidence (as measured by the log-Bayes factor in the top row of the plots) for a difference in conditional probabilities in the left column in both plots.

In all of the cohorts studied in Figs a, b, c, d and e, we report no evidence for the existence of dependencies between rounds that depart from a Markovian description (as can be inferred from the comparatively small log-Bayes factors for the blue and green conditional probabilities in the right column of all plots). This is an interesting, and

perhaps surprising, result regarding the nature of human behaviour in response to mass drug administration.

References

1. Boatin BA, Basáñez MG, Prichard RK, Awadzi K, Barakat RM, García HH, et al. A research agenda for helminth diseases of humans: towards control and elimination. PLoS neglected tropical diseases. 2012;6(4):e1547. Available from: https://doi.org/10.1371/journal.pntd.0001547.

2. Krentel A, Fischer PU, Weil GJ. A review of factors that influence individual compliance with mass drug administration for elimination of lymphatic filariasis. PLoS neglected tropical diseases. 2013;7(11):e2447. Available from: https://doi.org/10.1371/journal.pntd.0002447.

3. Truscott JE, Hollingsworth TD, Brooker SJ, Anderson RM. Can chemotherapy alone eliminate the transmission of soil transmitted helminths? Parasites & vectors. 2014;7(1):266. Available from: https://doi.org/10.1186/1756-3305-7-266.

4. Babu BV, Babu GR. Coverage of, and compliance with, mass drug administration under the programme to eliminate lymphatic filariasis in India: a systematic review. Transactions of the Royal Society of Tropical Medicine and Hygiene. 2014;108(9):538–549. Available from: https://doi.org/10.1093/trstmh/tru057.

5. Irvine MA, Reimer LJ, Njenga SM, Gunawardena S, Kelly-Hope L, Bockarie M, et al. Modelling strategies to break transmission of lymphatic filariasis - aggregation, adherence and vector competence greatly alter elimination. Parasites & Vectors. 2015 Oct;8(1):547. Available from: https://doi.org/10.1186/s13071-015-1152-3.

6. Coffeng LE, Bakker R, Montresor A, de Vlas SJ. Feasibility of controlling hookworm infection through preventive chemotherapy: a simulation study using the individual-based WORMSIM modelling framework. Parasites & vectors. 2015;8(1):541. Available from: https://doi.org/10.1186/s13071-015-1151-4.

7. Shuford KV, Turner HC, Anderson RM. Compliance with anthelmintic treatment in the neglected tropical diseases control programmes: a systematic review. Parasites & vectors. 2016;9(1):29. Available from: https://doi.org/10.1186/s13071-016-1311-1.

8. Farrell SH, Truscott JE, Anderson RM. The importance of patient compliance in repeated rounds of mass drug administration (MDA) for the elimination of intestinal helminth transmission. Parasites & Vectors. 2017 Jun;10(1):291. Available from: https://doi.org/10.1186/s13071-017-2206-5.

9. World Health Organization. Guideline: preventive chemotherapy to control soil-transmitted helminth infections in at-risk population groups. World Health Organization; 2017.

10. Fogarty L, Roter D, Larson S, Burke J, Gillespie J, Levy R. Patient adherence to HIV medication regimens: a review of published and abstract reports. Patient Education and Counseling. 2002;46(2):93 – 108. Available from: http://www.sciencedirect.com/science/article/pii/S0738399101002191.

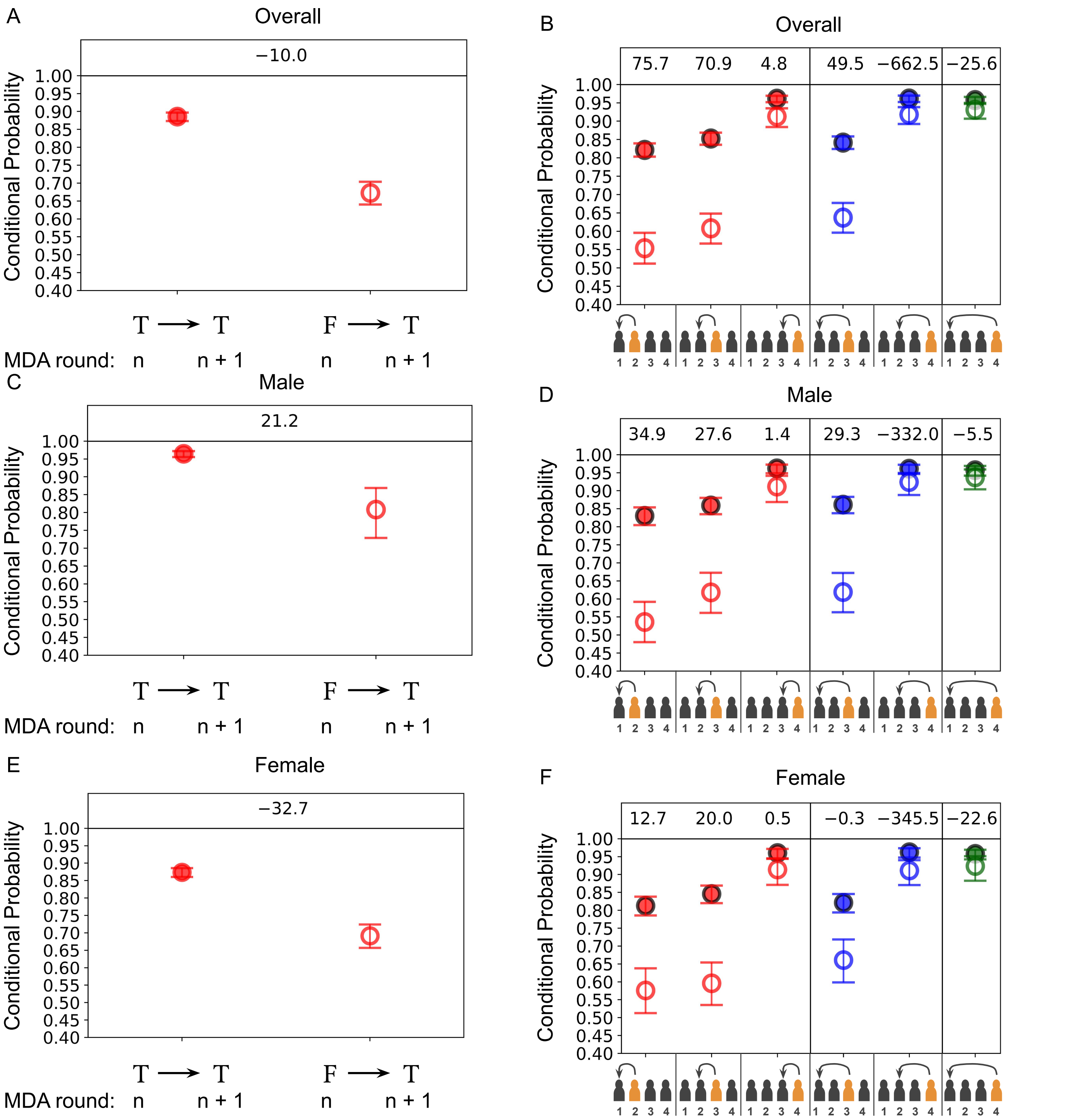

Fig a. *Left column:* The maximum likelihood as well as the limits of the marginalised 95% credible region for the conditional probabilities of receiving treatment for any given pair of sequential rounds (these are hence homogeneous in time and the process is Markovian) given treatment (filled points) or non-treatment (hollow points) in a previous round. *Right column:* The same as the left column but with allowed time dependence in the conditional probabilities of receiving treatment in each respective round (highlighted in orange on the horizontal axes). In each case the components corresponding to a given round were measured assuming all other respective rounds were inferred to be from time-dependent past behaviour-independent adherence and hence the likelihood is given in Appendix S1. Different colours for each point correspond to different lengths in time for the dependencies in behaviour. The datasets used are from the standard pre-SAC (0-4) age category from a cohort of individuals from the biannual treatment arm of the TUMIKIA project where the: top row corresponds to the overall group; middle row corresponds to the male sub-group; and bottom row corresponds to the female sub-group.

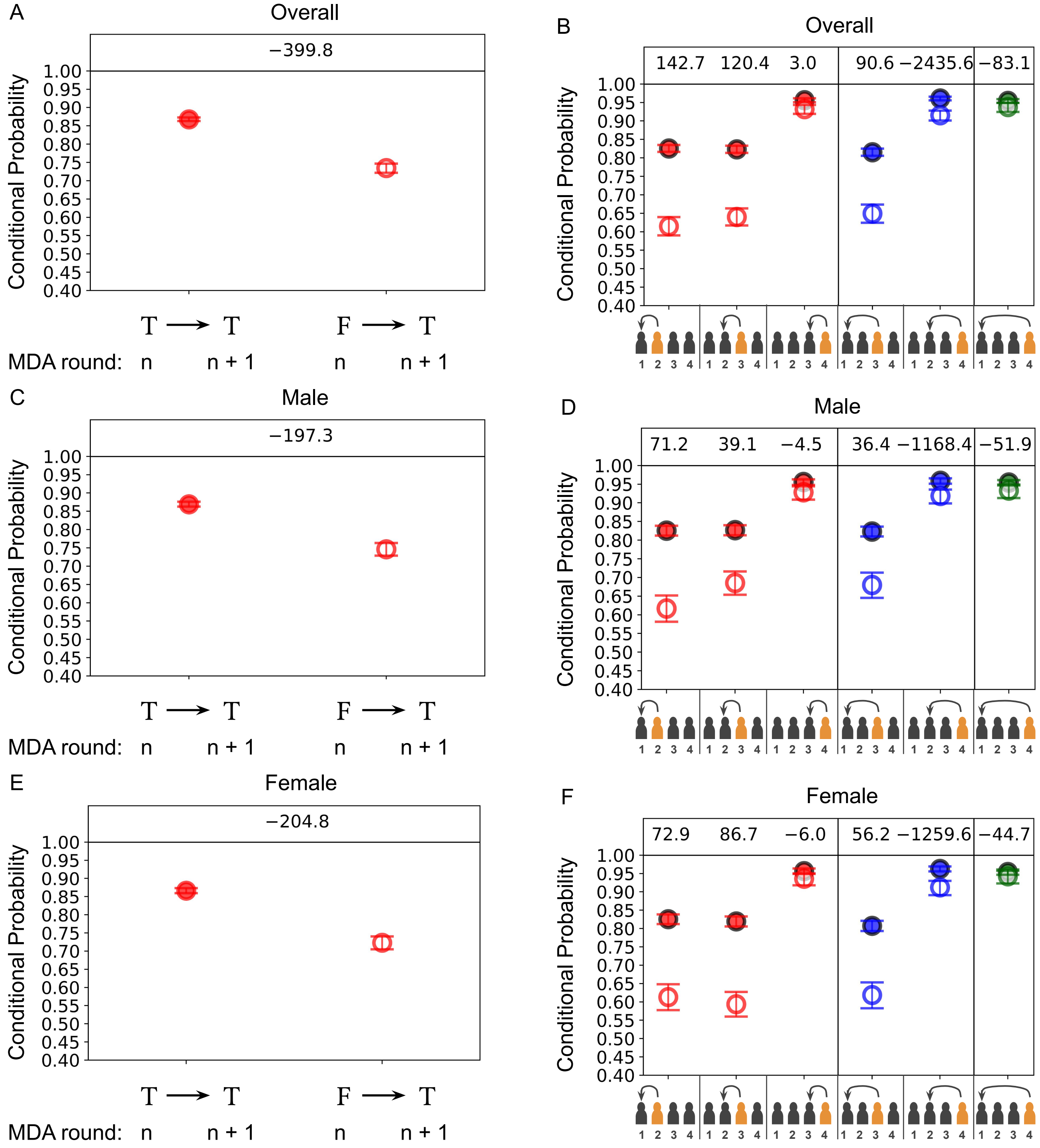

Fig b. *Left column:* The maximum likelihood as well as the limits of the marginalised 95% credible region for the conditional probabilities of receiving treatment for any given pair of sequential rounds (these are hence homogeneous in time and the process is Markovian) given treatment (filled points) or non-treatment (hollow points) in a previous round. *Right column:* The same as the left column but with allowed time-dependent in the conditional probabilities of receiving treatment in each respective round (highlighted in orange on the horizontal axes). In each case the components corresponding to a given round were measured assuming all other respective rounds were inferred to be from time-dependent past behaviour-independent adherence and hence the likelihood is given in Appendix S1. Different colours for each point correspond to different lengths in time for the dependencies in behaviour. The datasets used are from the standard SAC (4-15) age category from a cohort of individuals from the biannual treatment arm of the TUMIKIA project where the: top row corresponds to the overall group; middle row corresponds to the male sub-group; and bottom row corresponds to the female sub-group.

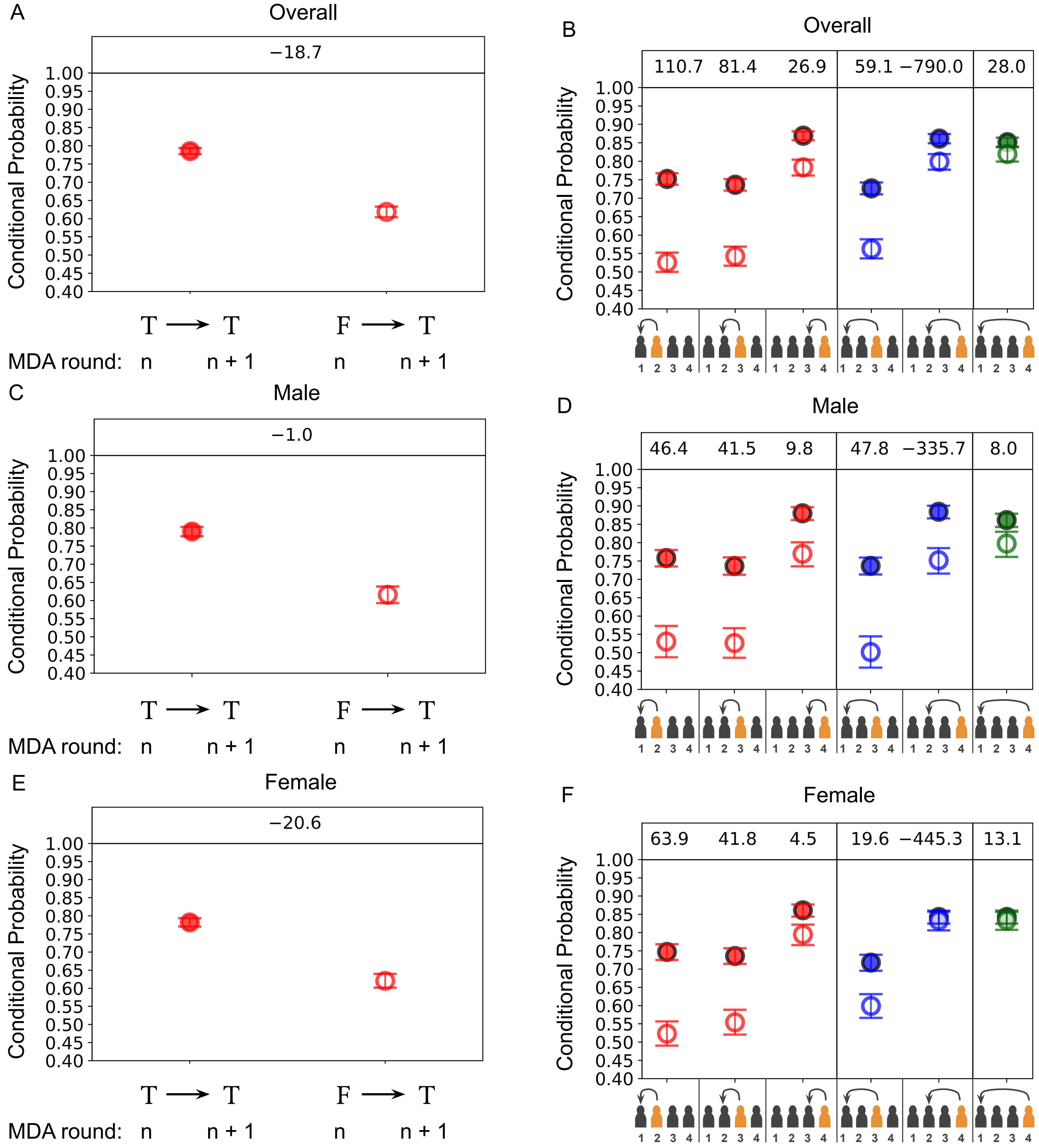

Fig c. *Left column:* The maximum likelihood as well as the limits of the marginalised 95% credible region for the conditional probabilities of receiving treatment for any given pair of sequential rounds (these are hence homogeneous in time and the process is Markovian) given treatment (filled points) or non-treatment (hollow points) in a previous round. *Right column:* The same as the left column but with allowed time-dependent in the conditional probabilities of receiving treatment in each respective round (highlighted in orange on the horizontal axes). In each case the components corresponding to a given round were measured assuming all other respective rounds were inferred to be from time-dependent past behaviour-independent adherence and hence the likelihood is given in Appendix S1. Different colours for each point correspond to different lengths in time for the dependencies in behaviour. The datasets used are from the 15-29 age category from a cohort of individuals from the biannual treatment arm of the TUMIKIA project where the: top row corresponds to the overall group; middle row corresponds to the male sub-group; and bottom row corresponds to the female sub-group.

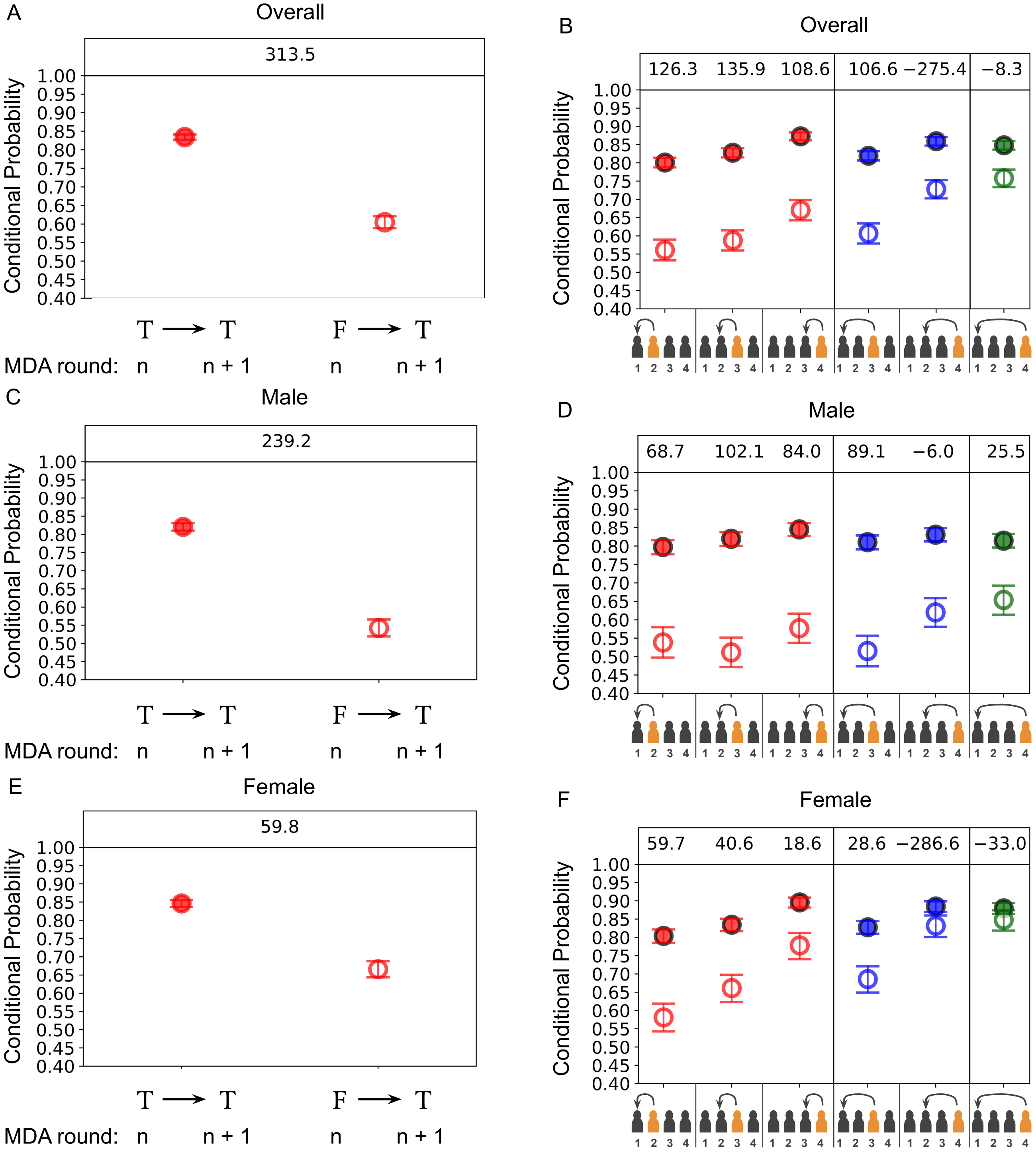

Fig d. *Left column:* The maximum likelihood as well as the limits of the marginalised 95% credible region for the conditional probabilities of receiving treatment for any given pair of sequential rounds (these are hence homogeneous in time and the process is Markovian) given treatment (filled points) or non-treatment (hollow points) in a previous round. *Right column:* The same as the left column but with allowed time dependence in the conditional probabilities of receiving treatment in each respective round (highlighted in orange on the horizontal axes). In each case the components corresponding to a given round were measured assuming all other respective rounds were inferred to be from time-dependent past behaviour-independent adherence and hence the likelihood is given in Appendix S1. Different colours for each point correspond to different lengths in time for the dependencies in behaviour. The datasets used are from the 30-49 age category from a cohort of individuals from the biannual treatment arm of the TUMIKIA project where the: top row corresponds to the overall group; middle row corresponds to the male sub-group; and bottom row corresponds to the female sub-group.

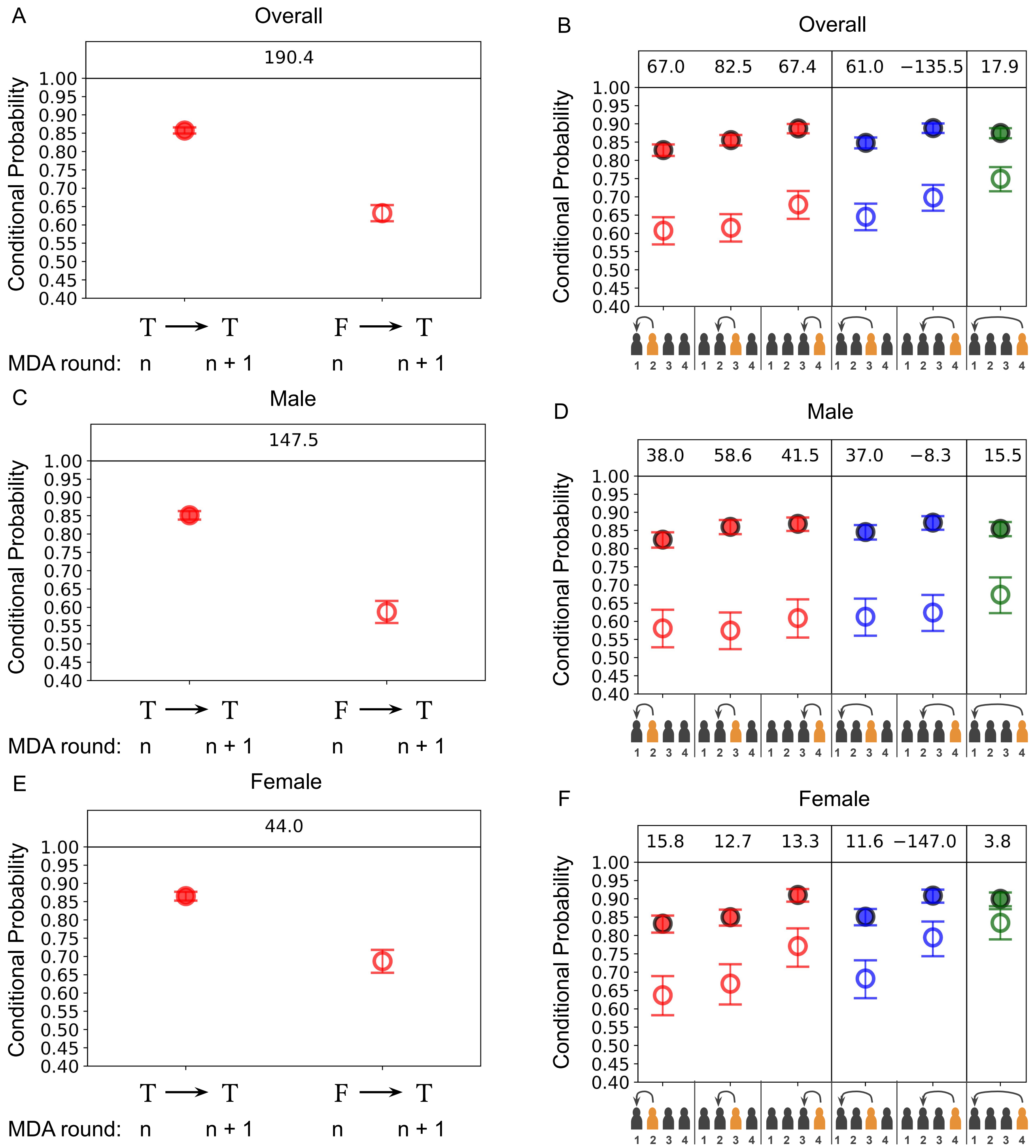

Fig e. *Left column:* The maximum likelihood as well as the limits of the marginalised 95% credible region for the conditional probabilities of receiving treatment for any given pair of sequential rounds (these are hence homogeneous in time and the process is Markovian) given treatment (filled points) or non-treatment (hollow points) in a previous round. *Right column:* The same as the left column but with allowed time dependence in the conditional probabilities of receiving treatment in each respective round (highlighted in orange on the horizontal axes). In each case the components corresponding to a given round were measured assuming all other respective rounds were inferred to be from time-dependent past behaviour-independent adherence and hence the likelihood is given in Appendix S1. Different colours for each point correspond to different lengths in time for the dependencies in behaviour. The datasets used are from the 50+ age category from a cohort of individuals from the biannual treatment arm of the TUMIKIA project where the: top row corresponds to the overall group; middle row corresponds to the male sub-group; and bottom row corresponds to the female sub-group.

11. Pullan RL, Halliday KE, Oswald WE, Mcharo C, Beaumont E, Kepha S, et al. Effects, equity, and cost of school-based and community-wide treatment strategies for soil-transmitted helminths in Kenya: a cluster-randomised controlled trial. The Lancet. 2019;393(10185):2039–2050. Available from: https://doi.org/10.1016/S0140-6736(18)32591-1.

12. Halliday KE, Oswald WE, Mcharo C, Beaumont E, Gichuki PM, Kepha S, et al. Community-level epidemiology of soil-transmitted helminths in the context of school-based deworming: Baseline results of a cluster randomised trial on the coast of Kenya. PLoS neglected tropical diseases. 2019;13(8):e0007427. Available from: https://doi.org/10.1371/journal.pntd.0007427.

13. Oswald WE, Kepha S, Halliday KE, Mcharo C, Witek-McManus S, Hardwick RJ, et al. Patterns of individual non-treatment during multiple rounds of mass drug administration for control of soil-transmitted helminths in the TUMIKIA trial, Kenya: a secondary longitudinal analysis. The Lancet Global Health. 2020;8(11):e1418–e1426. Available from: https://doi.org/10.1016/S2214-109X(20)30344-2.

14. Plaisier AP. Modelling onchocerciasis transmission and control. Erasmus University Rotterdam; 1996.

15. Plaisier AP, Stolk WA, van Oortmarssen GJ, Habbema JDF. Effectiveness of annual ivermectin treatment for Wuchereria bancrofti infection. Parasitology Today. 2000;16(7):298–302. Available from: https://doi.org/10.1016/s0169-4758(00)01691-4.

16. Jeffreys H. The theory of probability. OUP Oxford; 1998.

17. Truscott JE, Ower AK, Werkman M, Halliday K, Oswald WE, Gichuki PM, et al. Heterogeneity in transmission parameters of hookworm infection within the baseline data from the TUMIKIA study in Kenya. Parasites & vectors. 2019;12(1):442. Available from: https://doi.org/10.1186/s13071-019-3686-2.

18. Ásbjörnsdóttir KH, Ajjampur SSR, Anderson RM, Bailey R, Gardiner I, Halliday KE, et al. Assessing the feasibility of interrupting the transmission of soil-transmitted helminths through mass drug administration: The DeWorm3 cluster randomized trial protocol. PLOS Neglected Tropical Diseases. 2018 01;12(1):1–16. Available from: https://doi.org/10.1371/journal.pntd.0006166.

19. Mekete K, Ower A, Dunn J, Sime H, Tadesse G, Abate E, et al. The Geshiyaro Project: a study protocol for developing a scalable model of interventions for moving towards the interruption of the transmission of soil-transmitted helminths and schistosome infections in the Wolaita zone of Ethiopia. Parasites & vectors. 2019;12(1):1–12. Available from: https://doi.org/10.1186/s13071-019-3757-4.

20. Dyson L, Stolk WA, Farrell SH, Hollingsworth TD. Measuring and modelling the effects of systematic non-adherence to mass drug administration. Epidemics. 2017;18:56–66. Available from: http://www.ncbi.nlm.nih.gov/pubmed/28279457http://www.pubmedcentral.nih.gov/articlerender.fcgi?artid=PMC5340860.

21. Griffin JT, Hollingsworth TD, Okell LC, Churcher TS, White M, Hinsley W, et al. Reducing Plasmodium falciparum Malaria Transmission in Africa: A Model-Based Evaluation of Intervention Strategies. PLoS Medicine. 2010 aug;7(8):e1000324. Available from: http://dx.plos.org/10.1371/journal.pmed.1000324.
