# Supplementary material for: Individual adherence to mass drug administration in neglected tropical disease control: A probability model conditional on past behaviour
Source: PLoS Negl Trop Dis. 2021 Jan 22;15(1):e0009112. doi: 10.1371/journal.pntd.0009112 (PMC7857569; doi:10.1371/journal.pntd.0009112)
Supplement: S3 Appendix — (PDF) [file pntd.0009112.s003.pdf]

# S3 Appendix.

**Summary.** In this supplementary information, we analyse some of the existing models of adherence from the literature in the context of our proposed framework.

## The Plaisier model

Several models of MDA treatment programmes employ an adherence model developed by Plaisier in the context of onchocerciasis control [8, 20]. The Plaisier model assigns a propensity score for adherence to each individual which they then retain for the duration of the MDA programme [14, 15]. In the time-independent form, this model would be characterised by us as a heterogeneous population, time-independent model with no explicit individual dependence on past behaviour — though more recently developed models include time dependent effects through coverage variability between rounds. In the simplest model, the individual probability of adherence is given by $U^{(1-c)/c}$, where $U$ is a uniform random number and $c$ is expected probability of treatment and hence the expected coverage. The model is therefore completely parameterized by the overall expected coverage. The PDF for the adherence probability for this process is given by

$$\pi(p) = \frac{c}{1-c} p^{(2c-1)/(1-c)} . \tag{1}$$

The PDF of $p$ rises monotonically from zero to one for all values of $c > 0.5$ and falls monotonically for $c < 0.5$ (for $c = 0.5$, it is flat). Note that $\pi(p)$ is a beta distribution: $\pi(p) = \mathrm{Beta}[p; c/(1-c), 1]$. For this distribution, the mean failure run length is hence given by

$$\mathrm{E}(n_{\mathrm{F}}) = \frac{c}{2c-1} . \tag{2}$$

Note that in this model, adherence failure run length becomes undefined at a coverage of 50% or less. Additionally, one can show that the variance of this random variable becomes undefined for values of coverage below 66%, suggesting that failure run lengths in finite populations drawn from this distribution will exhibit extreme variability.

The probability of an individual being untreated across $N$ rounds of MDA in this model can also be calculated, giving

$$\pi_{\mathrm{un}} = \int_0^1 (1-p)^N \mathrm{Beta}[p; c/(1-c), 1] \, \mathrm{d}p = \frac{c}{1-c} B[c/(1-c), N+1] , \tag{3}$$

where $B(\cdot, \cdot)$ is the beta function. Fig a shows the distribution of adherence probabilities for 2 different coverage values and also the probability of an individual not adhering with treatment across a 4-round MDA programme.

Note here that in order to fully implement the Plaiser model in our framework, the additional variability in time through coverage would have to be added to our description. This should be straightforward to do within our proposed formalism.

## The Griffin Model

The adherence model used by Irvine et al [5] to model MDA adherence in the treatment of lymphatic filariasis was originally created by Griffin et al in the context of intervention strategies against malaria transmission [21]. The original Griffin model is

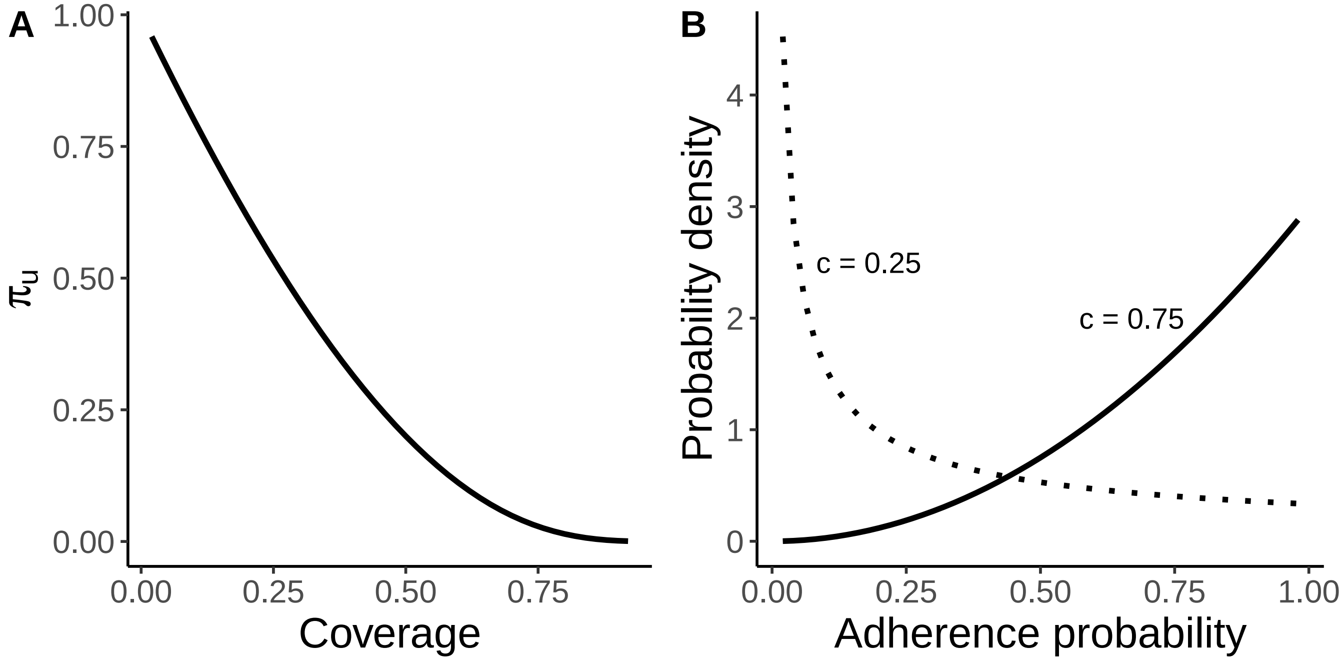


**Fig a.** A) Probability of an individual with adherence drawn from the Plaisier distribution of not adhering with treatment during a 4 round MDA programme. B) The probability distribution for adherence for coverages of 25% and 75%.

quite broad and deals with multiple simultaneous interventions and the correlations in their uptake. It does not include conditional dependencies for an individual's behaviour and is therefore a heterogeneous population, time-independent, individually past behaviour-independent model in its simplest form. Each individual in the population is assigned a correlation parameter, $u_i$, drawn from a normal distribution with mean $u_0$ and variance $\sigma^2$. These parameters are retained throughout the MDA programme. At each round a MDA round, each individual draws a unit-variance normal deviate with mean $u_i$, $z$. Treatment is received if $z < 0$. The expected coverage is given by $\phi(-u_0/\sqrt{1+\sigma^2})$, where $\phi$ is the standard normal cumulative probability function. This leaves one free parameter to control the distribution of adherence probabilities across the population.

The cumulative distribution of adherence probability, $p$, is given by

$$\pi(p) = \phi[\phi^{-1}(p;0,1) + u_0; 0, \sigma^2]\,, \tag{4}$$

giving a PDF

$$P(p) \propto \exp\left[-\frac{1-\sigma^2}{2\sigma^2}\left(\phi^{-1}(p) + \frac{u_0}{1-\sigma^2}\right)^2\right]\,. \tag{5}$$

The function $\phi^{-1}(p;0,1)$ varies monotonically in the range $(-\infty,\infty)$ with $p$. In Eq (5), the parameter $\sigma = 1$ acts to discriminate between two functional forms. For $\sigma < 1$, the distribution has a 'normal' shape with a single local maximum, while for $\sigma > 1$, the distribution has asymptotes with local maxima at the $p = 0$ and/or 1. In this, it is very similar, qualitatively, to the beta distribution (see Fig b).

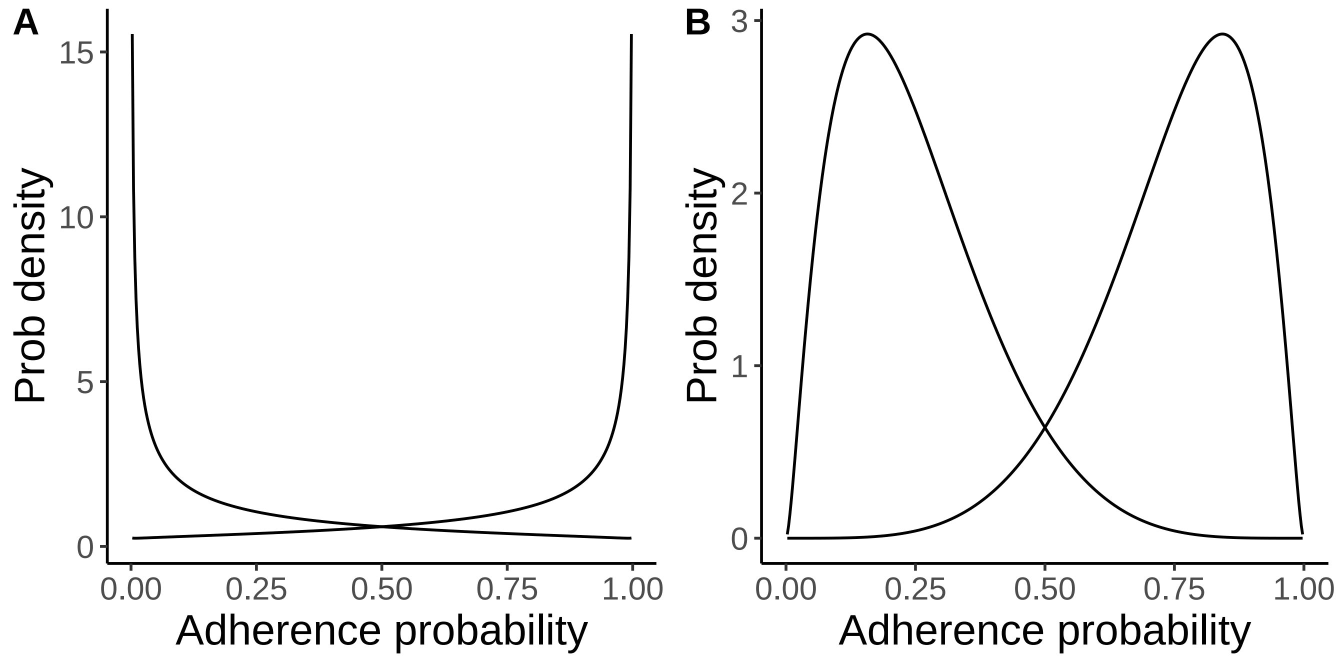


**Fig b.** Adherence probability distributions with A) $\sigma = 1.2$ and B) $\sigma = 0.5$ for mean coverages of 25% and 75%. The probability distribution for adherence for coverages of 25% and 75%.

## References


1. Boatin BA, Basáñez MG, Prichard RK, Awadzi K, Barakat RM, García HH, et al. A research agenda for helminth diseases of humans: towards control and elimination. PLoS neglected tropical diseases. 2012;6(4):e1547. Available from: `https://doi.org/10.1371/journal.pntd.0001547`.

2. Krentel A, Fischer PU, Weil GJ. A review of factors that influence individual compliance with mass drug administration for elimination of lymphatic filariasis. PLoS neglected tropical diseases. 2013;7(11):e2447. Available from: `https://doi.org/10.1371/journal.pntd.0002447`.

3. Truscott JE, Hollingsworth TD, Brooker SJ, Anderson RM. Can chemotherapy alone eliminate the transmission of soil transmitted helminths? Parasites & vectors. 2014;7(1):266. Available from: `https://doi.org/10.1186/1756-3305-7-266`.

4. Babu BV, Babu GR. Coverage of, and compliance with, mass drug administration under the programme to eliminate lymphatic filariasis in India: a systematic review. Transactions of the Royal Society of Tropical Medicine and Hygiene. 2014;108(9):538–549. Available from: `https://doi.org/10.1093/trstmh/tru057`.

5. Irvine MA, Reimer LJ, Njenga SM, Gunawardena S, Kelly-Hope L, Bockarie M, et al. Modelling strategies to break transmission of lymphatic filariasis - aggregation, adherence and vector competence greatly alter elimination. Parasites & Vectors. 2015 Oct;8(1):547. Available from: `https://doi.org/10.1186/s13071-015-1152-3`.

6. Coffeng LE, Bakker R, Montresor A, de Vlas SJ. Feasibility of controlling hookworm infection through preventive chemotherapy: a simulation study using the individual-based WORMSIM modelling framework. Parasites & vectors. 2015;8(1):541. Available from: https://doi.org/10.1186/s13071-015-1151-4.

7. Shuford KV, Turner HC, Anderson RM. Compliance with anthelmintic treatment in the neglected tropical diseases control programmes: a systematic review. Parasites & vectors. 2016;9(1):29. Available from: https://doi.org/10.1186/s13071-016-1311-1.

8. Farrell SH, Truscott JE, Anderson RM. The importance of patient compliance in repeated rounds of mass drug administration (MDA) for the elimination of intestinal helminth transmission. Parasites & Vectors. 2017 Jun;10(1):291. Available from: https://doi.org/10.1186/s13071-017-2206-5.

9. World Health Organization. Guideline: preventive chemotherapy to control soil-transmitted helminth infections in at-risk population groups. World Health Organization; 2017.

10. Fogarty L, Roter D, Larson S, Burke J, Gillespie J, Levy R. Patient adherence to HIV medication regimens: a review of published and abstract reports. Patient Education and Counseling. 2002;46(2):93 – 108. Available from: http://www.sciencedirect.com/science/article/pii/S0738399101002191.

11. Pullan RL, Halliday KE, Oswald WE, Mcharo C, Beaumont E, Kepha S, et al. Effects, equity, and cost of school-based and community-wide treatment strategies for soil-transmitted helminths in Kenya: a cluster-randomised controlled trial. The Lancet. 2019;393(10185):2039–2050. Available from: https://doi.org/10.1016/S0140-6736(18)32591-1.

12. Halliday KE, Oswald WE, Mcharo C, Beaumont E, Gichuki PM, Kepha S, et al. Community-level epidemiology of soil-transmitted helminths in the context of school-based deworming: Baseline results of a cluster randomised trial on the coast of Kenya. PLoS neglected tropical diseases. 2019;13(8):e0007427. Available from: https://doi.org/10.1371/journal.pntd.0007427.

13. Oswald WE, Kepha S, Halliday KE, Mcharo C, Witek-McManus S, Hardwick RJ, et al. Patterns of individual non-treatment during multiple rounds of mass drug administration for control of soil-transmitted helminths in the TUMIKIA trial, Kenya: a secondary longitudinal analysis. The Lancet Global Health. 2020;8(11):e1418–e1426. Available from: https://doi.org/10.1016/S2214-109X(20)30344-2.

14. Plaisier AP. Modelling onchocerciasis transmission and control. Erasmus University Rotterdam; 1996.

15. Plaisier AP, Stolk WA, van Oortmarssen GJ, Habbema JDF. Effectiveness of annual ivermectin treatment for Wuchereria bancrofti infection. Parasitology Today. 2000;16(7):298–302. Available from: https://doi.org/10.1016/s0169-4758(00)01691-4.

16. Jeffreys H. The theory of probability. OUP Oxford; 1998.

17. Truscott JE, Ower AK, Werkman M, Halliday K, Oswald WE, Gichuki PM, et al. Heterogeneity in transmission parameters of hookworm infection within the

baseline data from the TUMIKIA study in Kenya. Parasites & vectors. 2019;12(1):442. Available from: https://doi.org/10.1186/s13071-019-3686-2.

18. Ásbjörnsdóttir KH, Ajjampur SSR, Anderson RM, Bailey R, Gardiner I, Halliday KE, et al. Assessing the feasibility of interrupting the transmission of soil-transmitted helminths through mass drug administration: The DeWorm3 cluster randomized trial protocol. PLOS Neglected Tropical Diseases. 2018 01;12(1):1–16. Available from: https://doi.org/10.1371/journal.pntd.0006166.

19. Mekete K, Ower A, Dunn J, Sime H, Tadesse G, Abate E, et al. The Geshiyaro Project: a study protocol for developing a scalable model of interventions for moving towards the interruption of the transmission of soil-transmitted helminths and schistosome infections in the Wolaita zone of Ethiopia. Parasites & vectors. 2019;12(1):1–12. Available from: https://doi.org/10.1186/s13071-019-3757-4.

20. Dyson L, Stolk WA, Farrell SH, Hollingsworth TD. Measuring and modelling the effects of systematic non-adherence to mass drug administration. Epidemics. 2017;18:56–66. Available from: http://www.ncbi.nlm.nih.gov/pubmed/28279457http://www.pubmedcentral.nih.gov/articlerender.fcgi?artid=PMC5340860.

21. Griffin JT, Hollingsworth TD, Okell LC, Churcher TS, White M, Hinsley W, et al. Reducing Plasmodium falciparum Malaria Transmission in Africa: A Model-Based Evaluation of Intervention Strategies. PLoS Medicine. 2010 aug;7(8):e1000324. Available from: http://dx.plos.org/10.1371/journal.pmed.1000324.
